# A Solutions-Based Approach to Building Data-Sharing Partnerships

**DOI:** 10.5334/egems.236

**Published:** 2018-08-22

**Authors:** Sarah E. Wiehe, Marc B. Rosenman, David Chartash, Elaine R. Lipscomb, Tammie L. Nelson, Lauren A. Magee, J. Dennis Fortenberry, Matthew C. Aalsma

**Affiliations:** 1 Indiana University School of Medicine, US; 2 Ann and Robert H. Lurie Children’s Hospital of Chicago, US; 3 Yale University School of Medicine, US; 4 Marion County Public Health Department, US

**Keywords:** data sharing, partnerships, population health, electronic health records

## Abstract

**Introduction:**

Although researchers recognize that sharing disparate data can improve population health, barriers (technical, motivational, economic, political, legal, and ethical) limit progress. In this paper, we aim to enhance the van Panhuis *et al*. framework of barriers to data sharing; we present a complementary solutions-based data-sharing process in order to encourage both emerging and established researchers, whether or not in academia, to engage in data-sharing partnerships.

**Brief Description of Major Components:**

We enhance the van Panhuis *et al*. framework in three ways. First, we identify the appropriate stakeholder(s) within an organization (e.g., criminal justice agency) with whom to engage in addressing each category of barriers. Second, we provide a representative sample of specific challenges that we have faced in our data-sharing partnerships with criminal justice agencies, local clinical systems, and public health. Third, and most importantly, we suggest solutions we have found successful for each category of barriers. We grouped our solutions into five core areas that cut across the barriers as well as stakeholder groups: Preparation, Clear Communication, Funding/Support, Non-Monetary Benefits, and Regulatory Assurances.

Our solutions-based process model is complementary to the enhanced framework. An important feature of the process model is the cyclical, iterative process that undergirds it. Usually, interactions with new data-sharing partner organizations begin with the leadership team and progress to both the data management and legal teams; however, the process is not always linear.

**Conclusions and Next Steps:**

Data sharing is a powerful tool in population health research, but significant barriers hinder such partnerships. Nevertheless, by aspiring to community-based participatory research principles, including partnership engagement, development, and maintenance, we have overcome barriers identified in the van Panhuis *et al*. framework and have achieved success with various data-sharing partnerships.

In the future, systematically studying data-sharing partnerships to clarify which elements of a solutions-based approach are essential for successful partnerships may be helpful to academic and non-academic researchers. The organizational climate is certainly a factor worth studying also because it relates both to barriers and to the potential workability of solutions.

## Introduction

Traditional biomedical data gleaned from electronic health records (EHR) and insurance claims contribute to only 10 percent to 30 percent of the determinants of health [3]; however, because these data types are often more accessible, researchers rely primarily on EHR and claims data in studies of population health [4,5]. Adding non-traditional biomedical [6] and non-biomedical data such as vital statistics, public health surveillance data, criminal justice data, and social service utilization data could address gaps in capturing environmental exposure [3], social circumstances [3], and behavioral patterns [3] that significantly influence population health.

Although researchers recognize that sharing disparate data can improve population health, barriers limit progress [1,2,3,6,7]. Our research team, among others [8,9,10,11,12], conducts population-level epidemiology in vulnerable populations by using traditional and non-traditional biomedical and non-biomedical data. Our research team includes academicians and a public health practitioner who are experts in chronic disease care systems, informatics, epidemiology, public health, statistical methodology, justice data, stakeholder engagement, and clinical care. Understanding the value of community-based participatory research (CBPR), our research team has built strong stakeholder partnerships. CBPR is a collaborative approach to research that, in all phases of the research process, equitably engages community members, representatives of community organizations, and researchers [13]. While our research team does not conduct CBPR in the strictest sense, we aspire to the core CBPR principles of engaging, developing, and maintaining partnerships [13]. We have been able to overcome barriers in data sharing identified in the van Panhuis *et al*. framework [1] in order to assemble a rich longitudinal data set derived from local clinical systems, a health insurer, public health, and criminal justice agencies.

**Figure 1:**
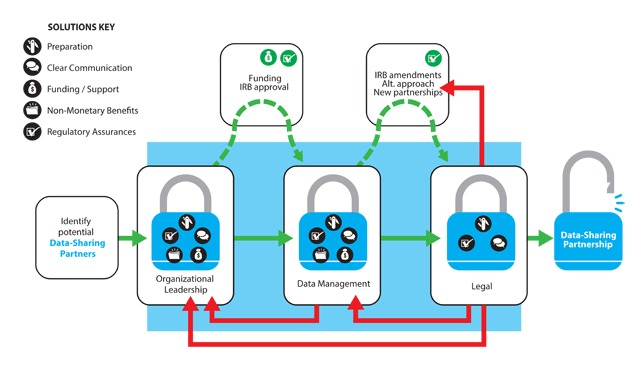
Solutions-Based Approach to a Data-Sharing Partnership: A Process Flow Diagram.

In the van Panhuis *et al*. data-sharing framework, the authors identified twenty common barriers and classified them into six categories: technical, motivational, economic, political, legal, and ethical [1]. Though van Panhuis *et al*. developed the framework with a focus on public health data, we found the barriers to be applicable to other data sources as well. In this paper, we aim to enhance the van Panhuis *et al*. framework and present a complementary solutions-based data-sharing process in order to encourage both emerging and established researchers, whether or not in academia, to engage in data-sharing partnerships. We primarily present an academic perspective; however, we believe that non-academic research teams can benefit from the enhanced framework and our experience. First, we provide an overview of the enhancements to the van Panhuis *et al*. framework (Table 1) and suggestions for using it when facing a barrier to sharing data. Then, we explain our complementary solutions-based process (Figure 1), with specific examples.

**Table 1:** Stakeholder Groups, Use Cases and Solutions Data-Sharing Partnerships by Barriers identified by van Panhuis *et al*. [1].

Category	Barrier	Stakeholder group	Use cases	Solutions

**Technical**	Data not collected Data not preserved Data not found Language barrier Restrictive data format Technical solutions not available Lack of metadata and standards	Data Management	Meaningful use implementation has changed what data are routinely collected in EHRs such as active medication lists, preferred language, smoking status for ≥13 year olds, etc. Certain surveillance databases maintain current clinical/public health records but not records of patients after death or out-migration from the geographic area of interest; many agencies change electronic data systems and lose data in the process (if some data elements are dropped or are not properly transferred). Language barriers can include spoken language/dialect or the jargon of a particular discipline which might present problems when applying similar methods or code across systems (e.g., identifying injury diagnoses within clinical, public health and/or justice data sources). Some agencies use paper rather than electronic data records and/or do not have the resources to transfer historical paper records into electronic format. Sharing data across disciplines often is difficult due to incompatible systems, making live data feeds, real-time record linkage, storage and computation of big data, and overall data-sharing difficult.	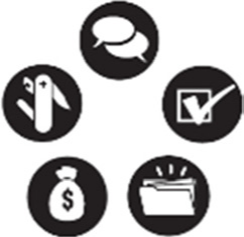
**Motivational**	No incentives Opportunity cost Possible criticism Disagreement on data use	Leadership Data Management Legal	Often overburdened data teams at non-clinical (and clinical) stakeholder organizations do not have the time or incentive to assist with external data requests; sometimes systems are not equipped to do or to process large data pulls, and effort or resources to overcome these hurdles outweigh perceived benefits. Data recipients may not understand the data received or the constraints of the data (e.g., data completeness or correct interpretation of individual data elements). Unawareness, misunderstanding, or lack of communication about how sharing data might benefit each of the stakeholder organizations.	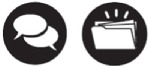
**Economic**	Possible economic damage Lack of resources	Leadership Data Management	Concern about possible discontent (or loss) of customers or about economic/emotional damage to customers if a data breach were to occur; these concerns are particularly acute when accessing sensitive or potentially stigmatizing data elements and/or after an unrelated data breach in one of the stakeholder organizations. Lack of staffing resources to provide data access and handle external data requests.	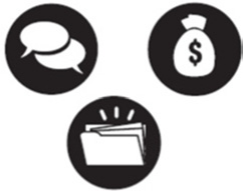
**Political**	Lack of trust Restrictive policies Lack of guidelines	Leadership Legal	Restriction in documenting adolescent clinical encounters or mental health/behavioral health diagnoses because of privacy laws or other privacy concerns. Stigma associated with increased diagnosis rates (e.g., HIV or syphilis) among certain populations and how these rates will be perceived or will have differential impact.	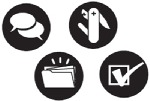
**Legal**	Ownership and copyright Protection of privacy	Leadership Legal	Some data schemas and other preliminary information necessary to identify data needs and processes are restricted or require additional agreements. Many agencies are bound by substantial restrictions in data-sharing, and often it is not clear to outsiders what the processes are for requesting data, or what the standards are for analytic use and storage of data extracts; lack of clarity can precipitate extra time-consuming discussions with multiple stakeholders and sub-stakeholders.	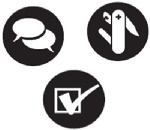
**Ethical**	Lack of proportionality Lack of reciprocity	Leadership Legal	Concern for sharing juvenile court records because of their sensitive nature, and questions about whether the potential impact of use of these data outweighs the potential risks. Inadequate *a priori* communication about how credit for the work is to be distributed among the participants (or participating entities).	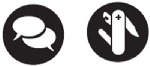
**Solutions Key**				
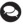 Clear Communication				
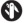 Preparation				
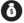 Funding/Support				
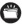 Non-Monetary Benefits				
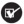 Regulatory Assurances				

## Brief Description of Major Components

### Enhanced framework

We enhance the van Panhuis *et al*. framework in three ways (Table 1). First, we identify the appropriate stakeholder(s) within an organization (e.g., criminal justice agency) with whom to engage in addressing each category of barriers (Table 1). We typically engage with three types/groups of stakeholders within an organization: the leadership team, the data manager or data management team, and the legal department designee or team. Often one individual fills more than one of these roles. Second, we provide concrete use cases. These use cases are a sample of specific challenges that we have faced in our data-sharing partnerships with criminal justice agencies, local clinical systems, and public health. Third, and most importantly, we suggest solutions that we have found successful for each category of barriers. We grouped our solutions into five core areas that cut across the barriers as well as stakeholder groups: preparation, clear communication, funding/support, non-monetary benefits, and regulatory assurances. We based the solutions on a combination of practical experience and CBPR facilitating factors: provision of financial and other incentives, and partnership skills/competencies such as communication, listening, conflict resolution, negotiation, and capability to collaborate with multicultural organizations with different power structures [13]. By ‘preparation,’ we mean identifying potential key stakeholders (organizations and individuals within organizations) and understanding how their goals and objectives comport with the research aims, prior to the first meeting [13]. By ‘clear communication [13],’ we mean competency in communication, listening, conflict resolution, and negotiation, and the ability to understand and collaborate with multicultural organizations with different power structures. We also seek to document at subsequent meetings that we have made progress on follow-up tasks/deliverables, and to be flexible when challenges arise. When we are prepared, our research team conveys respect for the potential (or existing) stakeholder’s time and a high level of commitment to the aspiring (or existing) partnership. By ‘funding/support,’ we mean financial and in-kind support to stakeholders for data access. By ‘non-monetary benefits,’ we mean products (or services) provided to stakeholders not related to data access. These solutions help build strengths and resources within the stakeholders’ organization and provide financial and other incentives to collaborate [13,14]. By ‘regulatory assurances,’ we mean oversight or protections to reduce and/or appropriately share risk associated with exchanging data.

It is our intent that researchers may reference Table 1 to help them determine the appropriate solution(s) to employ and the appropriate stakeholder(s) within a data-sharing organization to engage when facing a specific barrier. For example, if the data-sharing organization changes electronic data systems resulting in a ‘data not found’ technical barrier, one of the appropriate solutions is a ‘clear communication’ strategy with the stakeholder’s data manager or data management team to learn more about the situation.

### Data-sharing process model

Our solutions-based process model (Figure 1) complements Table 1 by detailing the appropriate order in which to engage potential/existing stakeholders before starting a research project. An important feature of the process model is the nonlinear, iterative process. Usually, interactions begin with the leadership team and progress to both the data management and legal teams, moving forward (green arrows in Figure 1) or backward (red arrows in Figure 1) to readdress barriers or address new ones, with additional solutions identified and offered to stakeholders.

**Identify and learn more about potential partners.** We have identified potential data-sharing stakeholders (organizations and individuals within an organization) at various stages of the research process. Ideally, our research team identifies potential stakeholders early in the research process so we can collectively develop and refine the research question(s) [13,14]. Our research team may identify a potential stakeholder through our own online research, as well as through word of mouth from either colleagues or existing stakeholders. Since these personal contacts convey trust, they have helped our research team significantly in engaging new stakeholders. Conversely, potential stakeholders have approached our research team to collaborate on project development, evaluation, or grant writing. These stakeholder-initiated partnerships can offer exciting research opportunities as well. For example, an emergency medical services (EMS) director approached our team to analyze criminal arrests involving drug seizures and subsequent drug overdose deaths in the same vicinity. He hypothesized that an interruption in the drug supply chain may lead to increased risk of drug overdose. Moreover, our research team connects with potential (and existing) stakeholders through clinical service. For example, members of our research team provide health care and implement a behavioral health-screening program at a juvenile detention center. Through providing this clinical service to the juvenile court system, our research team gained intimate knowledge of the juvenile court system’s processes and needs. Furthermore, our research team established a trusted relationship that has developed into research projects using highly sensitive, identifiable juvenile detention stay records. In addition, engagement on community- and state-based boards can build connections with organizational leaders and their social networks. Being a member of a board not only benefits the community; new partnerships may emerge.

**Engage with organizational leadership.** The stakeholder’s organizational leadership may include one or multiple contacts. In our experience, multiple contacts provide several potential avenues of engagement if one stakeholder within the organization either does not respond or quickly dismisses the request. Our preparation for engaging with the stakeholder may be iterative as we seek new types of information at different phases of data-sharing development. Our research team prepares by studying the potential stakeholder’s organizational mission, leadership structure, current partners, data availability, prior history of data-sharing, and overall organizational needs. We anticipate potential barriers (e.g., funding/support, legal, data security) based on the stakeholder’s organizational history (e.g., history of a data breach) and prior data-sharing partnerships. When possible, we familiarize ourselves with the stakeholder’s specific data use guidelines or with the general data use guidelines within the stakeholder’s discipline. For example, data security requirements may be different for health (i.e., Health Insurance Portability and Accountability Act or HIPAA) than for education (i.e., Family Educational Rights and Privacy Act or FERPA) data. If such information is not readily available, we make sure to discuss these topics during an introductory meeting with the stakeholder’s leadership. Our preparation in advance of this meeting communicates our interest to the stakeholder and establishes a degree of engagement up front. Regardless of our research team’s preparation, we begin by listening to understand the potential (or existing) stakeholder’s needs. Even with the most extensive preparation, often our research team discovers that the stakeholder’s priorities or emphases, and/or the distinct views held by particular stakeholders within the organization differ from what we expected. For example, often stakeholders will be interested in how the other data sources can better describe the population they serve such as by identifying the number of pregnancies among adolescents following release from detention or by understanding what proportion of people living with HIV have mental health and substance use diagnoses within the clinical system. In addition, we encounter some differences in terminology, so a part of our process is learning each stakeholder’s language. In these ways, our research team strives to develop a shared understanding of a stakeholder’s needs and our own research goals. We find that clear communication leads to a positive outcome for both sides in the data-sharing partnership. In addition, our research team seeks a stakeholder’s input on how our research might meaningfully benefit its population, which strengthens the relevancy of the research question, study design, and public dissemination of findings.

Once our research team has clarity about how our research goals can advance the stakeholder’s mission, we proceed to discuss our research project. At this phase, we often learn about the stakeholder’s apprehensions about sharing data, such as legal barriers (e.g., risk of a data breach) and economic barriers (Table 1). First, our research team acknowledges the potential risks in sharing data and the potential benefits also. If relevant, our research team proactively discloses past data security mistakes by our university and/or discusses high profile data security mistakes of other organizations. We identify how these mistakes and experiences have informed the protections that we currently have in place. Because some of our potential stakeholders are unaware of our university’s robust resources, we inform them about and invite them to consider using our infrastructure, such as the institutional review board (IRB) process, legal department consultation, dedicated data server, data security measures, standard data use agreements, and other regulatory processes. After the meeting, we leave an appropriately detailed synopsis of the university’s infrastructure for further review and for reference at subsequent meetings with the stakeholder’s leadership or legal counsel. During subsequent discussions regarding agreement documents, we make note of the regulatory assurances that may help alleviate concerns about how we will use the data. Our data use agreements articulate who bears the burden or risk [15], address individual privacy and data security [15], and provide a clear description of the scope of work. We allow our partner stakeholders to review in advance any public presentation or publication to ensure that we handle sensitive (“political”) issues tactfully and to add to our safeguards against inadvertently disclosing personal health information (PHI) or other sensitive information. Furthermore, although we often engage with multiple stakeholder organizations simultaneously, we negotiate separate agreements with each organization [15].

Second, a stakeholder’s leaders often relay to us their concern about the economic barriers (Table 1) associated with sharing data. From the stakeholder’s perspective, the resources necessary to share data might outweigh the benefits of the potential partnership. Our research team prepares in advance for this by determining the type of support (e.g., funding, in-kind support, non-monetary benefits) we can offer. In addition to providing financial support for the stakeholder’s data management team (or other relevant expenditure), we may provide non-financial support that creates mutual benefits for the stakeholder as well as our research team (e.g., providing workers and/or expertise to transfer data from obsolete servers, or to transcribe written records into electronic format). We have offered access to the shared/linked data and our research team’s data management and analysis capabilities, either for stakeholder-initiated grant applications or for other needs. For example, many of our criminal justice stakeholders appreciate when we create a summary/descriptive report of our shared data for them to use in grant applications because they do not have the resources to create it themselves. Our research team considers this type of work as an investment and valuable for us regardless of the direct relationship to the research aims because it builds trust. Our research team also offers to handle as many of the bureaucratic steps of data-sharing as possible such as reviewing data and data schemas prior to data transfer, drafting the data use agreement, and managing the data transfer process (or other prerequisite steps). We start with the stakeholder’s easily solvable challenges then proceed incrementally to more difficult challenges, as shared experience and trust increase. Our research team shares the results and the infrastructure developed during the project, when possible, to increase the capacity of our stakeholders and to sustain our partnership. Ideally, our stakeholders are sufficiently involved throughout the research process to be included as co-authors on publications, rather than simply signing off at the end of the project. For example, our research team studies the HIV continuum of care in populations with criminal justice involvement. Early in this program of research, we recognized the importance of partnering with a public health stakeholder with HIV surveillance data expertise in order to define the HIV clinical care quality measures and understand the context of the services provided. She was an integral member of our team, contributing to new research questions and writing both manuscripts and subsequent grant applications.

Once we have engaged with the stakeholder’s leadership, a possible next step (as indicated by the dashed green arrow in the Figure 1) is for our team to seek research funding (with a strong letter of support from the stakeholder’s leadership) and/or university IRB approval. Sometimes, we engage with the stakeholder’s data management team prior to seeking funding and/or university IRB approval (see below), although this may not be necessary, depending on the nature of our research project and the partnership.

**Engage with data management team.** Although the stakeholder’s leadership provides insights about the organization’s databases, the stakeholder’s data management team often has more precise and up-to-date information on the topic. In our experience, the stakeholder’s data management team is vital in data-sharing partnerships. Nevertheless, because of organizational and financial considerations of the stakeholder’s leadership, our research team often does not contact the data management team until after the leadership has signed-off or until after adequate resources are in place to support our research project. Sometimes we may even experience difficulty connecting with the stakeholder’s data management team because the stakeholder’s leadership protects them to focus on internal priorities. Once we engage with the data management team, we employ the same solutions as with the stakeholder’s leadership — preparation, clear communication, funding/support, and non-monetary benefit(s) to navigate barriers. When we communicate with the data management team to identify mutual benefit, we discover different needs than those expressed by the leadership. We candidly talk about the stakeholder’s data, the data manager’s role, and the financial and infrastructure needs to complete our research project. We glean a wealth of information about the stakeholder’s technical issues such as inadequate servers, obsolete data sources, which data types in the schema have higher or lower quality, and the constraints or limitations of the data. Therefore, we strive to develop a shared understanding of our research project and the stakeholder’s data and expertise so that we can avoid pitfalls in our use of these data and in reporting our research findings. Data completeness is a critical characteristic for determining the quality and utility of EHR data for research [16]. For example, in a data-sharing partnership with a large insurance stakeholder, our research team determined that claims data were not useful because of poor data completeness.

By understanding the role of the stakeholder’s data management team and its members’ expertise, our research team can determine what role(s) our university and our own research team members can play in the data access, transfer, management, and analysis process. For example, our research team finds that providing the stakeholder’s data management team with data quality checks, geocoded data, linked individual identifiers, or other data-related resources helps the stakeholder’s team complete its work. More broadly, in the course of a data-sharing partnership, if our research team can help our stakeholders identify and explain weaknesses (and strengths) in their data that were previously unknown or not understood, the work invested in one research project may benefit future projects. Through data-sharing our research team has unique opportunities to examine the “raw” data from multiple sources about a given individual, which enhances our understanding and helps us relay to our stakeholders information we’ve discerned about data quality and completeness. For example, as our research team evaluated the local health department’s HIV surveillance data, we identified a substantial number of missing records because of in- and out-migration of people living with HIV in the jurisdiction. As a result, our team, including the practitioner from the local public health department, pursued additional funding to “fix” the surveillance database issue and to enhance our knowledge about the impact of migration on HIV care outcomes in the region.

Our research team attempts to review the stakeholder’s data schema or data dictionary at an early stage, particularly when multiple databases are available, so we know how these databases interact and what each contains. Our review informs us about the relevant data elements for our research aims. (Occasionally, the stakeholder’s leadership considers the schema or data dictionary to be proprietary information and therefore requires an agreement or other assurances in advance.) Our team also prefers to invest some time and resources in reviewing an identifiable sample of the data or, in some cases, a de-identified sample with all elements to more definitively identify which elements are necessary and valuable for the research project (e.g., because of so much incompleteness, some fields have limited value).

Our research team attempts to reduce our stakeholder’s workload for our proposed data-sharing projects. For example, to reduce the burden upon the stakeholder’s data management team, we request full data tables rather than specific data elements within various tables. We are careful to balance these considerations with the priorities of the stakeholder’s leadership and legal department to ameliorate risk, to share the minimum necessary data, and to maintain data privacy.

After interacting with the data management team, sometimes our research team has to amend the IRB protocol by specifying additional data elements and data-related procedures. Likewise, when our research team identifies significant data quality gaps, we may have to pursue additional stakeholders and/or alternative data sources.

**Engage with legal team.** From our perspective, engaging with our university’s legal department and the stakeholder’s legal department is a key step in creating mutually acceptable data-sharing agreements. Communication and preparation, as when interacting with other members of the stakeholder’s organization, are key steps in overcoming barriers. Prior to pursuing data-sharing partnerships, our research team asks the university’s legal counsel to provide insight on potential constraints from the university’s standpoint, to identify templates for data use agreements, and to anticipate potential barriers. When our university’s legal counsel anticipates a significant barrier to data-sharing, our research team invites the university’s counsel to discuss data-sharing directly with the stakeholder’s legal counsel in order to reduce the need for multiple conversations (with the principal investigator on our research team as an intermediary). Moreover, our research team prepares to discuss in detail with the university and stakeholder legal departments the specific data elements needed and to justify that the elements represent the minimum necessary data to address the research aims. Likewise, our research team has a specific plan regarding all data-sharing procedures (i.e., data access at the stakeholder’s organization, data transfer to our university) as well as data use (i.e., data security, data access among our research team, publication and public presentation review plans). When engaging with the stakeholder’s legal department, our research team brings a sample data use agreement to the meeting that clearly articulates the burden of risk and scope of work as a guide for the conversation. Our stakeholders perceive this proactive step as helpful and a demonstration of our preparation and attention to detail. Furthermore, we find that standard agreements can expedite the data-sharing process (rather than drafting one from scratch), assuming the standard agreement is relevant from a legal risk standpoint. Therefore, our research team is careful to be knowledgeable about relevant data use guidelines specifically for our stakeholder and/or the stakeholder’s discipline (e.g., FERPA for education, HIPAA for health).

In our experience, the entire process of interacting with various stakeholders, particularly the legal team, can be very time consuming. Therefore, we incorporate adequate time in our grant applications and our personal/study team timelines. Furthermore, we include adequate funding for stakeholder-related resources in our grant applications, which is helpful when our research team needs to pursue data-sharing partnerships with alternative stakeholders to achieve our research aims. Presenting creative solutions to problems and leveraging CBPR principles have facilitated introductions to alternative partners in some cases (Figure 1). For example, in a recent four-year research project, we experienced significant delays in sharing data with a stakeholder because of its legal and regulatory processes that lasted over 18 months. During this process, however, we established strong ties with our institutional HIPAA compliance program such that we are a resource for other investigators in sharing similar data and in establishing procedures not previously available for our own more expedited data access in the future.

## Conclusions and Next Steps

Leveraging non-traditional biomedical data and non-biomedical data, along with EHR and claims data, for a holistic picture is essential to population health research [6]. Data sharing is a powerful tool in this research endeavor, but significant barriers hinder data-sharing partnerships [1]. By aspiring toward CBPR principles of engaging, developing, and maintaining partnerships [13], we have overcome barriers to data sharing and have achieved success with various data-sharing partnerships. In this paper, we shared a solutions-based process model to encourage and guide others, whether or not in academia, to engage in data-sharing partnerships.

Because a number of barriers may arise, data-sharing projects are rarely linear. Even when a research team implements each of the solutions (clear communication, preparation, funding/support, non-monetary benefits and regulatory assurances), some data-sharing projects may not come to fruition. Although disappointing, team members should not lose hope. By continuing to aspire toward CBPR principles like interacting in a respectful manner with the leadership and others in the organization, viable partnerships may emerge.

As a next step, it would be helpful to systematically study data-sharing projects to clarify which elements of a solutions-based approach are particularly important for successful partnerships. Moreover, the organizational climate is a factor worth studying because it relates both to barriers and to the viability of solutions.

In sum, a CBPR framework can be helpful in understanding how best to partner with agencies for data-sharing purposes [14]. A core feature of a CBPR framework is building collaborative partnerships in all research phases. Our team has found that long-term and sustainable partnerships are what yield the most rewarding and impactful data-sharing research.
